# The epidemic characteristics and spatial autocorrelation analysis of hand, foot and mouth disease from 2010 to 2015 in Shantou, Guangdong, China

**DOI:** 10.1186/s12889-019-7329-5

**Published:** 2019-07-24

**Authors:** Haoyuan Zhang, Lianpeng Yang, Liping Li, Guangxing Xu, Xubin Zhang

**Affiliations:** 1 MPH Education Center and Injury Preventive Research Center, Shantou University Medical College, 22 Xinling Road, Shantou, 515041 China; 2 Zhuhai Maternal and Child Health Care Hospital, 543 Ningxi road, Zhuhai, 519001 China; 3 Shantou Center for Disease Control and Prevention, 58 Shanfen road, Shantou, 515041 China

**Keywords:** Hand, foot and mouth disease (HFMD), Epidemic characteristics, Spatial autocorrelation analysis, Shantou

## Abstract

**Background:**

Hand, foot and mouth disease (HFMD) is the highest incidence of infectious diseases in China. Shantou is one of the most infected cities. Therefore, it is necessary for us to understand the epidemic characteristics and distribution trend of HFMD in Shantou. The purpose of this study is to investigate the spatial epidemiological characteristics of HFMD and analyse its spatial autocorrelation.

**Method:**

We collated and summarised the data of HFMD in Shantou from 2010 to 2015. SaTScan software and Moran’s I were used to analyse the spatial correlation of HFMD, and the results were presented in ArcMap.

**Results:**

The distribution of HFMD in Shantou was of a seasonal trend, mainly concentrating during May and June. Children under 5-years-old were the main group of cases of HFMD, accounting for 92.46%. The proportion of infected children, especially those aged zero to 1, was the largest in each year, accounting for 45.62%, meaning that smaller children were more susceptible to HFMD. The number of male patients with HFMD was greater than that of females (1.78:1, male: female). With regard to the potential impact of patients’ living style on the incidence rate of HFMD, this study revealed that scattered children were the dominant infected population, accounting for as much 84.49% of cases. The incidence of HFMD was unevenly distributed among streets. The incidence interval of streets was in a range of 13.76/100,000 to 1135.19/100,000. Spatial autocorrelation analysis showed that there was no global spatial correlation in Shantou, except in 2013. The results of local spatial autocorrelation analysis showed that H-H correlation existed in the high incidence local area of Shantou.

**Conclusions:**

The incidence of HFMD across the various streets in Shantou not only varied widely but also represented local autocorrelation. Attention, as well as prevention and control measures, should be focused on those high-incidence areas, such as the Queshi street, Zhuchi street and Xinjin street.

## Background

Hand, foot and mouth disease (HFMD) is a common infectious disease caused by enterovirus of the highly infectious picornaviridae, and infectious viruses are primarily based on coxsackievirus A16(Cox-A16) and enterovirus 71(EV71) [1–4]. The major susceptible population of HFMD is infants and children who are under 5-years-old [5]. The clinical manifestations of the patients are herpes and eczema in the hands, feet, mouths and hips. Most HFMD patients can be cured by symptomatic treatment, but a small number of children with HFMD are subject to more complicated diseases, such as myocarditis, pulmonary edema, aseptic meningitis and even death [6]. HFMD is a considerable global public health challenge, especially in the Asia-Pacific region [7]. South Korea [8], Thailand [9], Vietnam [10] and other Asian regions once erupted HFMD in different scales and to different degrees [9]. During January 2004 and December 2013, the infectious diseases with the highest annual incidence were HFMD in China [11]. Besides treating the infected, the government should focus on the prevention and control of HFMD. Previous studies of HFMD principally focused on the seasonal effects of meteorological factors [12, 13], the determinants of the national changes in the reported cases [12], the spatial and temporal aggregation of the region [14], the development and efficacy of the vaccine [15, 16], and analyses of the clinical features, diagnosis [17], virology and pathogenesis [7].

As an analytical method, spatial autocorrelation analysis has been widely used in economic and social sciences [18]. Spatial autocorrelation refers to the potential dependence of some variables on the observed data in the same distribution area. In recent years, many scholars have applied space technology to various diseases, including dengue [19] and tuberculosis [20]. There were also some studies on the use of spatial autocorrelation analysis for HFMD [21]. Xie [22] used spatiotemporal scanning statistics to explore the spatial-temporal aggregation of HFMD in Guangxi Zhuang Autonomous Region. Deng [23] analysed the spatial and temporal clustering of HFMD in Guangdong Province, and the spatial scale was limited to the district and county level. Fei [24] applied space-time scanning statistics to establish a real-time simulation testing system from provincial and district levels, and earlier warning results were limited to provinces and counties. Compared with the traditional method, spatial autocorrelation analysis is used to analyse the spatial epidemic characteristics of disease distribution, and the correlation of incidence rate in adjacent regions can be obtained. Spatial autocorrelation analysis can provide suggestions for the government to formulate and implement appropriate regional public health intervention strategies to prevent and control HFMD. However, the larger the scale of research, and the larger the result coordinately, which will not cause financial waste, as well as wasted manpower and material resources for prevention work. Thus, the rational allocation and key distribution of resources could not be realised. According to the relevant research, the smaller the spatial scale, the more accurate prevention could be achieved [1, 23, 24]. At present, the street scale is the minimum scale to study the spatial aggregation of HFMD. However, few researchers have analysed the spatial clustering of HFMD based on street scale. To understand the incidence of HFMD in Shantou, this paper studied HFMD from 2010 to 2015 and revealed the distribution of HFMD in Shantou. At the same time, this study used Moran’s I statistic for research indicators and analysed the characteristics of geographic information cases of HFMD to explore the global spatial autocorrelation and local spatial autocorrelation of the incidence of HFMD among different streets in Shantou from 2010 to 2015.

## Methods

### Study area

As one of the special economic zones and important coastal cities in southeastern coastal areas, Shantou, located in Guangdong Province, is also a central city in eastern Guangdong. Lying in the jurisdiction of six municipal districts and one county, the total area is 2064 km^2^, with a resident population of is 5,579,200 until 2016. Shantou has a large resident population and a small floating population, but no research has been done on the spatial and temporal distribution characteristics of HFMD in this city. Street-scale is the minimum appropriate scale to study spatial clustering. Therefore, it is used as streets as the geographical unit of spatial analysis.

### HFMD data

The Ministry of Health of China listed HFMD as a national class C infectious disease in 2008 [25]. According to the state regulations, for the diagnosis of HFMD cases, the responsible reporter should fill in the infectious disease report card, and the responsible reporting unit should organise the staff to input report information. In the first few years, the Centers for Disease Control and Prevention (CDC) found that underreporting of HFMD frequently occurred because the diagnosis mechanism was incomplete. From 2008 to a certain period afterwards, the overall number of HFMD cases in Shantou has not been fully represented by the China Information System for Disease Control and Prevention. Therefore, we chose HFMD cases data from 2010 to 2015 as the research data. All the HFMD case data were from Shantou CDC. Shantou CDC authorised us to analyse HFMD data. The numbers of HFMD cases from 2010 to 2015 in Shantou were 4810, 10304, 9995, 8405, 11297 and 5550 respectively, with 50,361 cases in total. We excluded cases where the family address was not in the city of Shantou, and where the street address was unclear.

### Population data

The total number of permanent residents in Shantou was taken from the Shantou statistical yearbook. The total population in each street was calculated through the total number of street residents the year before divided by the total number of permanent residents and multiplied by the total number of permanent residents in that year.

### Statistical analysis

In this study, we used SaTScan software to analyze the spatial autocorrelation and Moran’s I [2, 3], after which we applied K-Nearest Neighbors (KNN) method to establish a spatial weight matrix and calculate the global spatial autocorrelation index for Moran’s I, as well as the local spatial autocorrelation index (local indicators of spatial association, LISA, *α* = 0.05). Global spatial autocorrelation is a description of the spatial characteristics of attribute values throughout the region. Local spatial autocorrelation is used to calculate the significant level of LISA, and local Moran’s I statistic is used to analyze the spatial difference degree between each region and the surrounding region. The analysis steps are as follows: the spatial weight matrix is established by the KNN method:, ${\mathbf{W}}_j=\frac{1}{{\boldsymbol{d}}_{ij}^{\mathrm{m}}}$.

m is the power, and d_*ij*_ represents the distance between region i and region j. The global spatial autocorrelation index, Moran’s I, is then calculated. There are n area units in the study area, and the observed values on the I unit are X_i_. The mean value of the observation variable in the N unit is $\overline{X}$. *W*_*ij*_ is a spatial weight matrix. Thus, Moran’s I is defined as:

$$ I=\frac{n\sum \limits_{i=1}^n\sum \limits_{j=i}^n{\boldsymbol{W}}_{ij}\left({X}_i-\overline{X}\right)\left({X}_j-\overline{X}\right)}{\left(\sum \limits_{i=1}^n\sum \limits_{j=i}^n{\boldsymbol{W}}_{ij}\Big)\right)\sum \limits_{i=1}^n{\left({X}_1-\overline{X}\right)}^2} $$

The value of Moran’s I statistics is between [− 1, 1]. *I* > 0 shows that there is a positive spatial correlation between research objects (the incidence of streets), which means 0 is irrelevant, while *I* < 0 shows negative spatial correlation.

LISA is used to reflect a geographical phenomenon on a regional unit or the degree of correlation between an attribute value and the same geographical phenomenon or attribute value on the adjacent unit. In this study, LISA is used to reflect the correlation degree between the incidence of a certain street and the incidence of a nearby street. The local Moran’s I index is defined as: ${I}_i=\frac{n\left({X}_i-\overline{X}\right)\sum \limits_{j=i}^n{\boldsymbol{W}}_{ij}\left({X}_j-\overline{X}\right)}{\sum \limits_{i=1}^n{\left({X}_i-\overline{X}\right)}^2}$.

In the formula, *n* is the number of space units involved in the analysis.

*X*_*i*_ and *X*_*j*_ represent the observational values of a phenomenon (or an attribute characteristic) *x* on the *i* and *j* of the space unit. W_*ij*_ is the spatial weight.

If *I*_*i*_ = 0, there is no spatial autocorrelation. This shows that there is no aggregation around the area, implying a random distribution; if *I*_*i*_ < 0, there is a spatial negative correlation; if *I*_*i*_ > 0, there is a positive spatial correlation. The greater the absolute value of *I*_*i*_, the higher the aggregation degree around the area. When the *I*_*i*_ value is positive, it is a high incidence area. When *I*_*i*_ is negative, it is an area of low incidence.

There are four types of local spatial connection forms between the regional unit and its adjacent area units: high value and high value (H-H), low value and low value (L-L), high value and low value (H-L), low value and high value (L-H) [26].

## Results

### Demographic characteristics of patients

The majority of patients with HFMD in Shantou were mainly infants (under 1-year-old) and young children. Children less than 1-year-old were the main victims of the disease, accounting for 45.62% of the total incidence of the disease. The proportion of patients with HFMD under 5-years-old in Shantou took up 92.46% of the total incidence of the disease. The HFMD incidence of males is higher than that of females, and the ratio of male to female case numbers is 1.78:1. Among the different groups, the scattered children are the main body of the disease. The number of cases, turning out to be 84.49%, is the largest in the total population, which is greatly higher than that of the other groups (Table 1).

**Table 1:** 2010–2015 demographic characteristics of patients with HFMD in Shantou

		2010	2011	2012	2013	2014	2015	Total	Percentage(%)
Age	~ 1	2012	4460	4661	4498	4453	2790	22874	45.62
	~2	1253	2829	2489	1851	2809	1293	12524	24.98
	~ 3	671	1542	1533	1029	1865	827	7467	14.89
	~ 4	318	669	679	496	1046	295	3503	6.99
	~ 5	146	348	240	207	501	146	1588	3.17
	> 5	194	456	393	324	623	199	2189	4.37
Gender	Male	3103	6837	6688	5103	6883	3480	32094	64.00
	Female	1491	3467	3307	3302	4414	2070	18051	36.00
Type	Scattered Children	3505	8960	9130	7635	8551	4586	42367	84.49
	Kindergarten Children	949	1064	636	577	2396	844	6466	12.89
	Pupil	124	248	177	156	308	105	1118	2.20
	Other	16	32	52	37	42	15	194	0.39

### The trend of HFMD

**Fig. 1:**
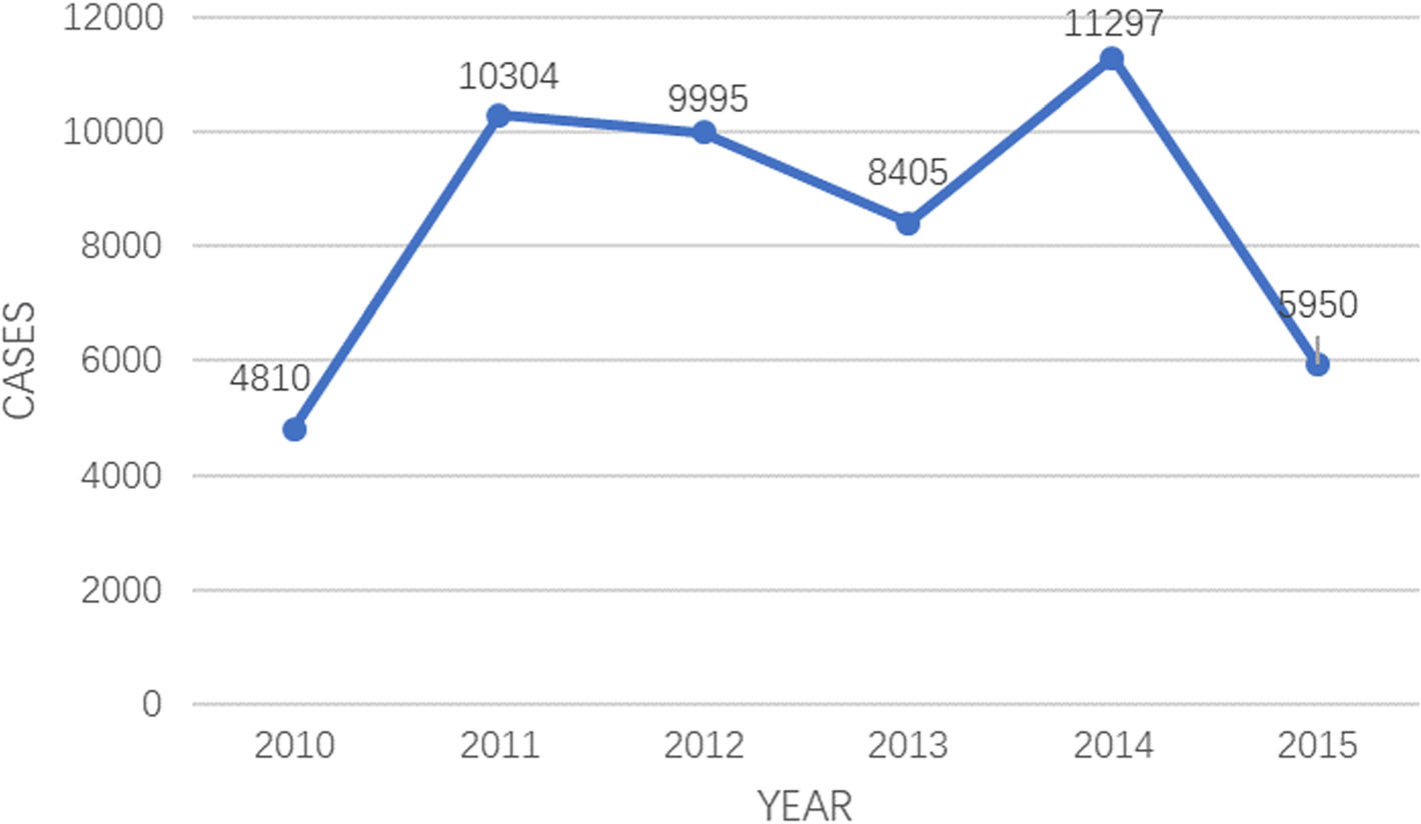
Changes in the number of HFMD in 2010–2015 in Shantou

**Fig. 2:**
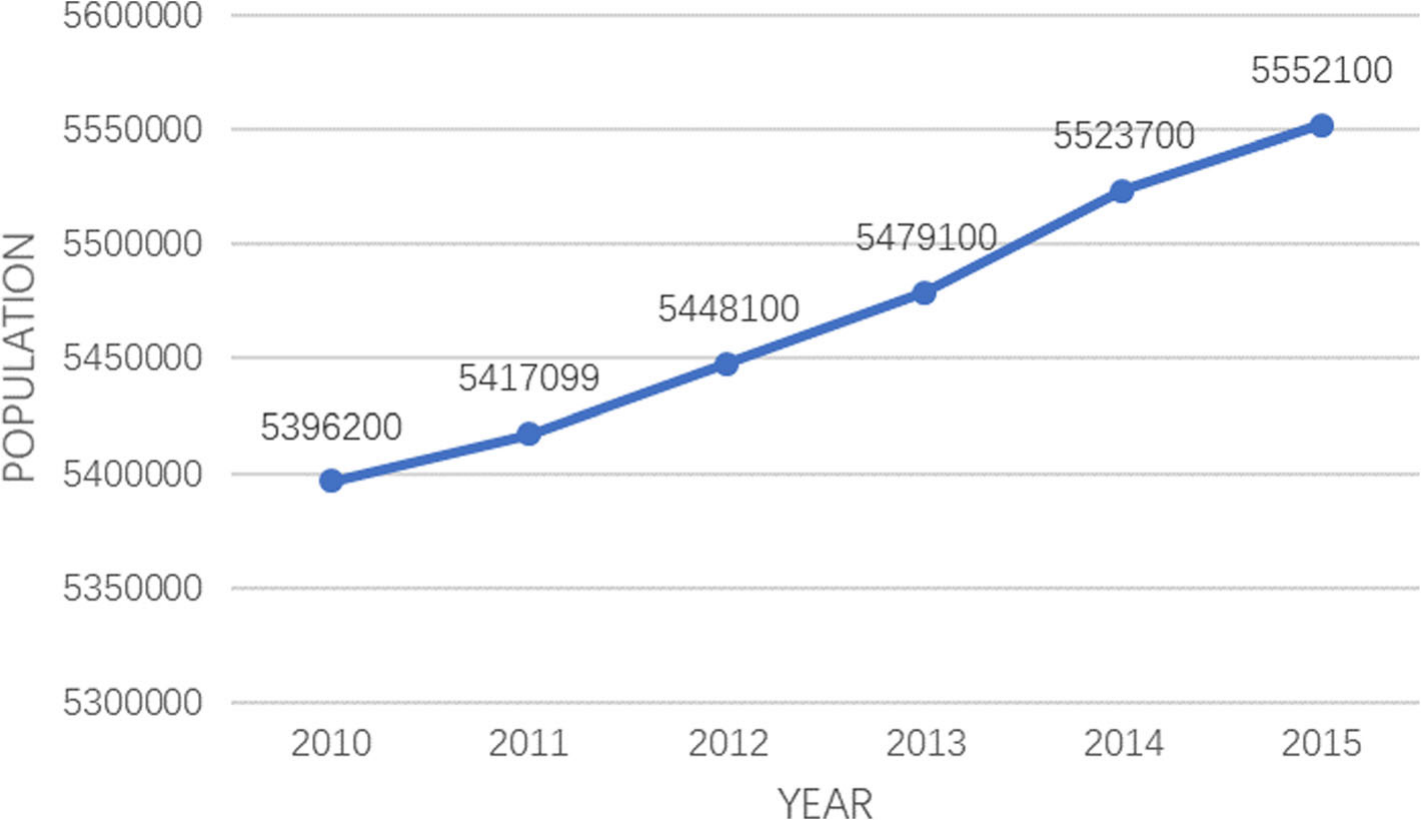
Changes in the number of the population in 2010–2015 in Shantou

From Fig. 1, we could see that from 2010 to 2015, the number of cases in Shantou first climbed, and then dropped. The highest number of cases was 11297 in 2014. By 2015, the incidence of HFMD in Shantou sharply declined, and the number of HFMD cases was about half of that in 2014, around 5950. In Fig. 2, we could see the population change of Shantou. From Figs. 1 and 2, we could know that the incidence of HFMD from 2010 to 2015 were respectively 89.13/100,000, 190.21/100,000, 183.46/100,000, 153.40/100,000, 204.52/100,000 and 107.17/100,000. The incidence of HFMD was the highest in 2014 and the lowest in 2010.

### Time distribution of HFMD

**Fig. 3:**
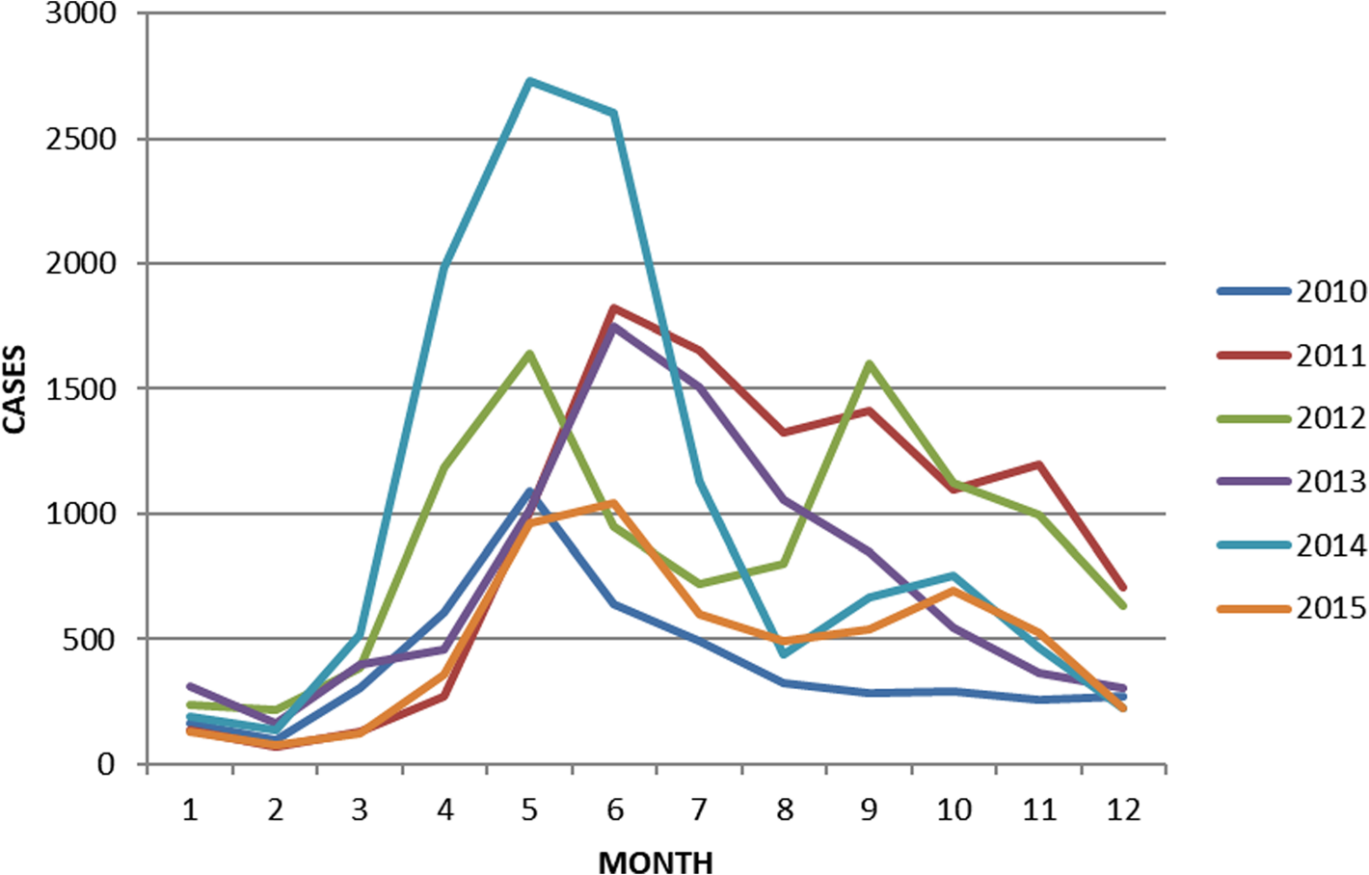
Time distribution of HFMD in 2010–2015 in Shantou

From Fig. 3, it is clear that HFMD in Shantou was found all year around. The incidence of HFMD in Shantou has a mainly unimodal distribution, and the peak incidence concentrated in May and June. The trend of bimodal distribution was not significant. In the past 6 years, the bimodal distribution only occurred in 2012, and the peak incidence was in May and September respectively.

### Spatial distribution of HFMD

There are 69 streets in Shantou, the incidence rate of HFMD varies among these streets. From 2010 to 2015, the street incidence ranged from 13.76/100,000 to 1135.19/100,000, and the incidence varied widely.

The area with the highest incidence was Xinjin street in 2014 with an incidence rate of 1135.19/100,000. While the lowest incidence appeared in Hongchang town in 2010 with a prevalence of 13.76/100,000.

**Fig. 4:**
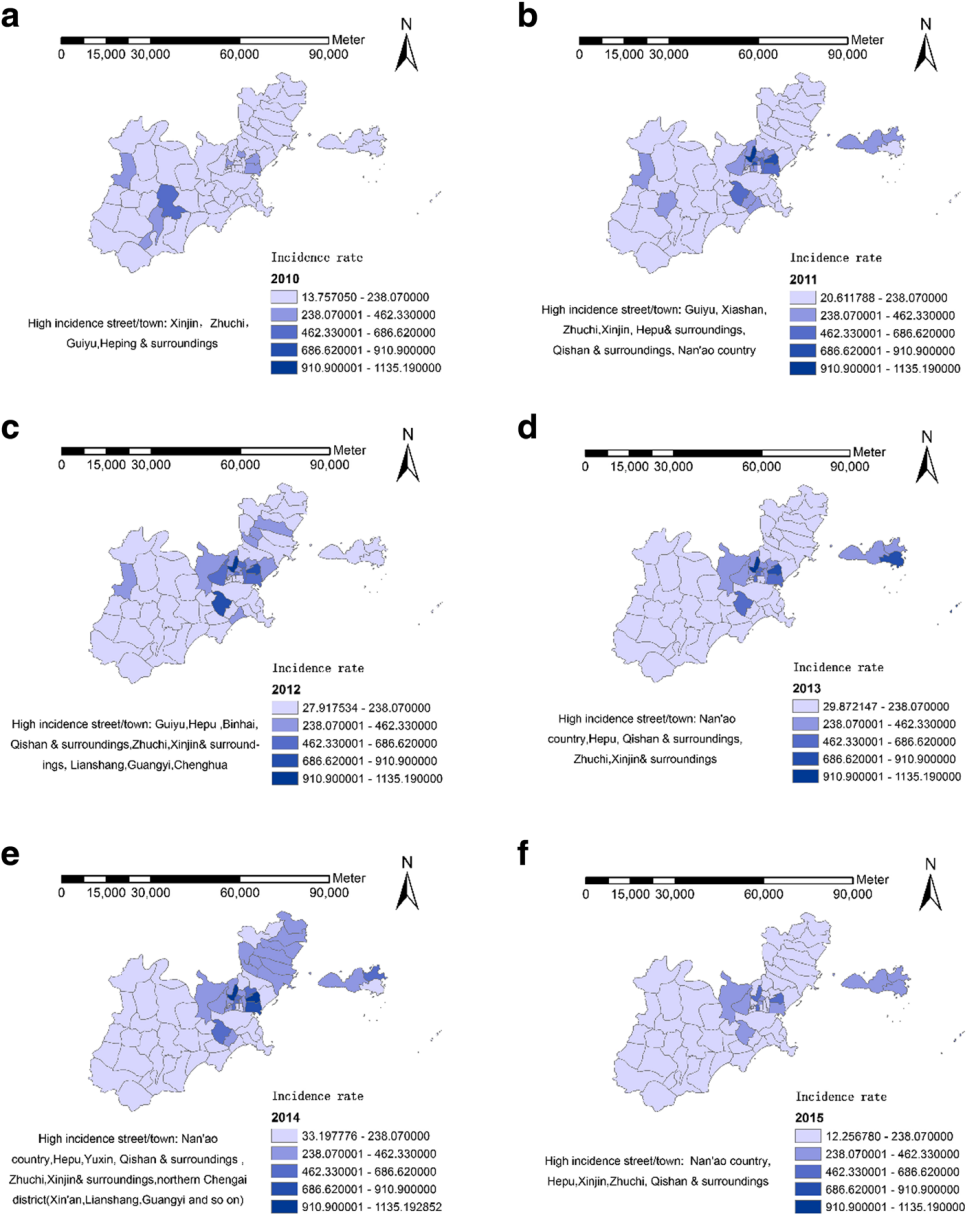
2010–2015 (**a**-**f**) Shantou HFMD high incidence area

**Fig. 5:**
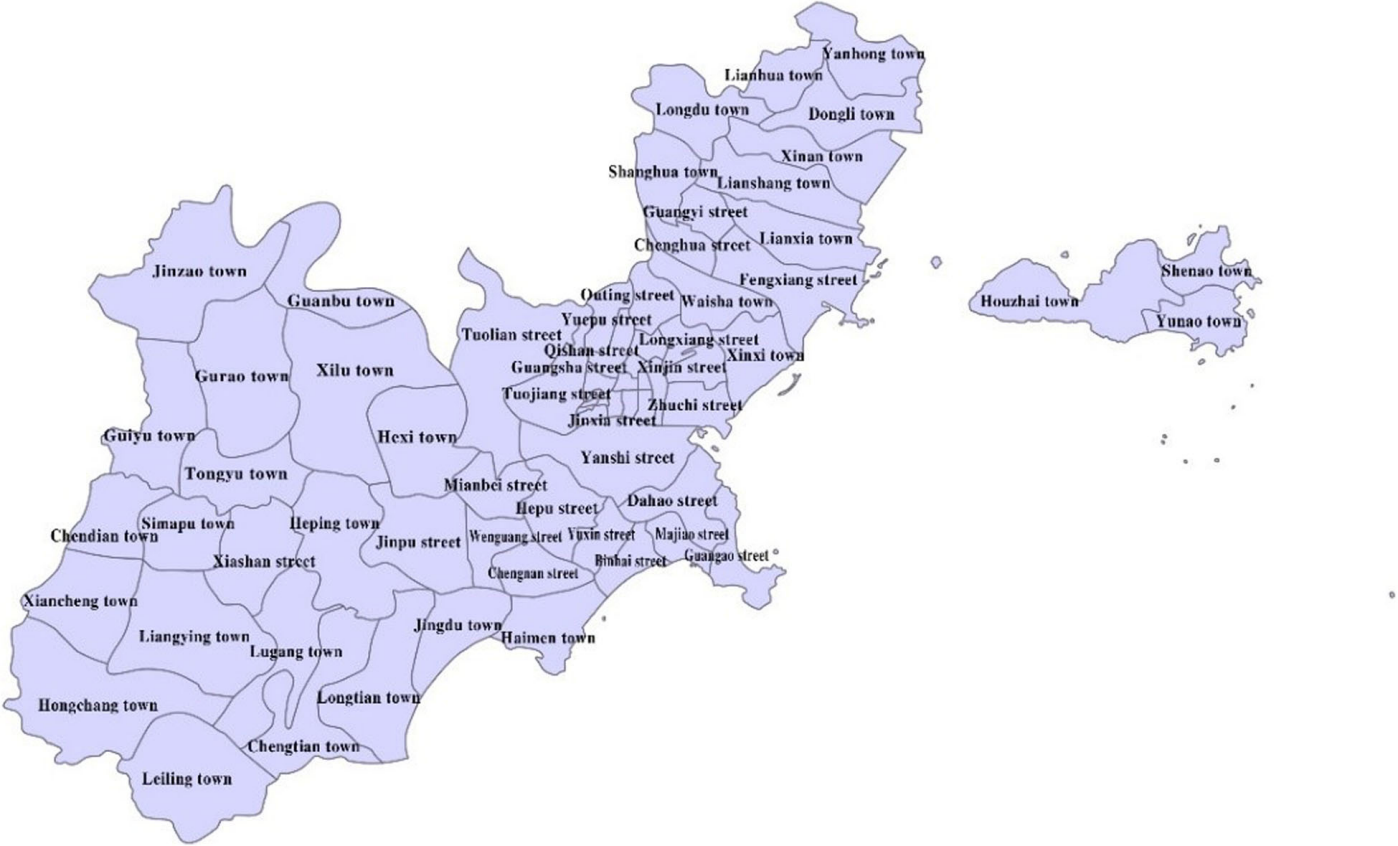
Name of different streets/towns of Shantou city

From the Figs. 4 and 5, we could know that high incidence areas mainly include Zhuchi street, Hepu street, Xinjin street, Qishan street and other streets nearby. The other high incidence areas were mainly found in the northern Chenghai district and Nan’ao county.

As shown in Figs. 4 and 5:

### Global autocorrelation analysis

The global Moran’s I index of HFMD in Shantou from 2010 to 2015 was 0.0934, 0.0582, 0.0587, 0.0934, 0.0426 and 0.0557. Except for 2013, when *P*-value was less than 0.05, the other years’ *P*-value was more than 0.05. There was no statistical significance in this difference. This has shown that the HFMD in Shantou had global autocorrelation only in 2013. In 2013, the spatial distribution of HFMD showed a non-random state with spatial aggregation and a certain global spatial positive correlation.

### Local autocorrelation analysis

**Fig. 6:**
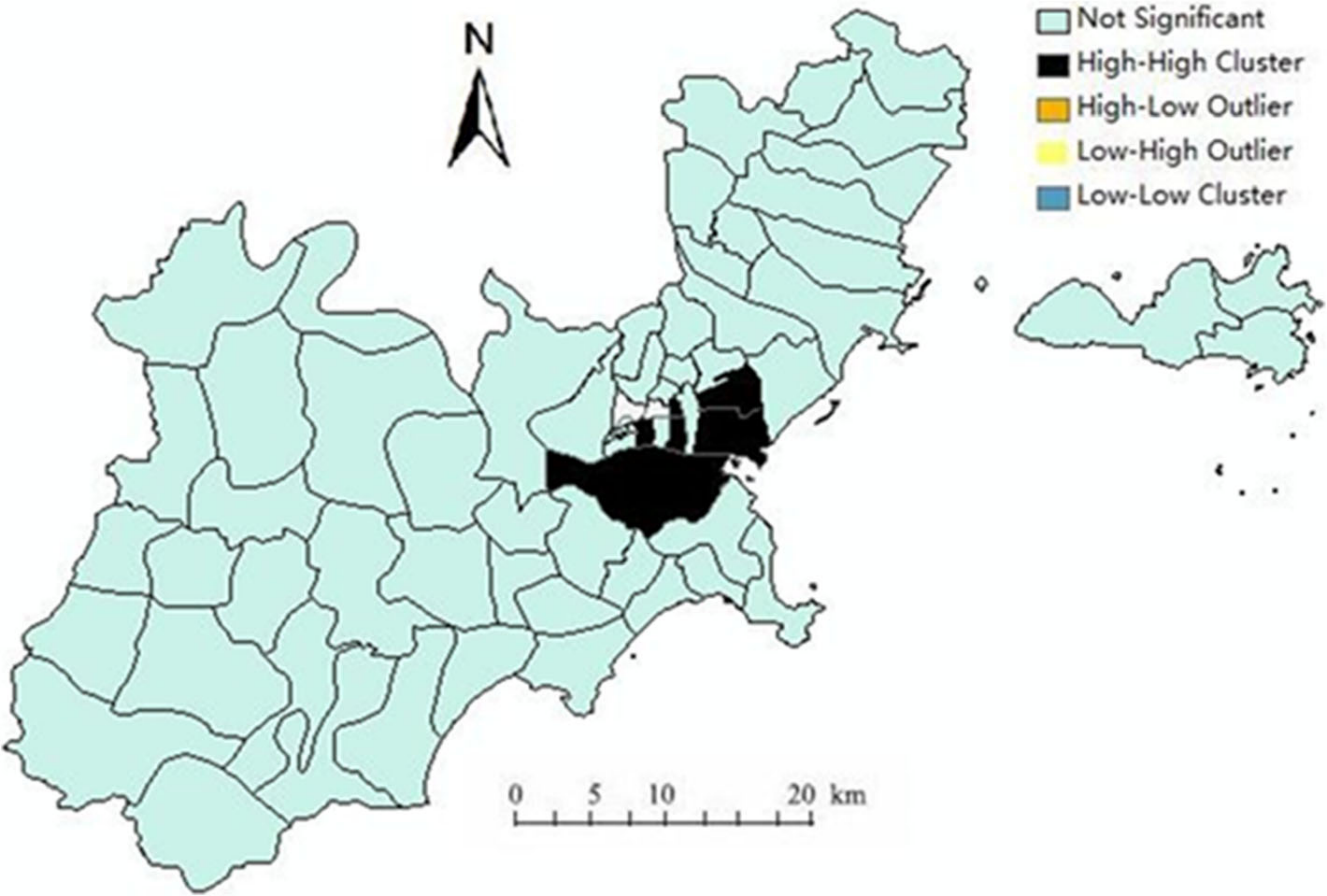
Results of local spatial autocorrelation analysis in Shantou in 2013

In 2013, the H-H gathering areas in Shantou mainly concentrated in the Queshi street, Xinjin street and Zhuchi street. The incidence of the other streets around these three streets was also high. As shown in Fig. 6:

## Discussion

Here we studied the data of HFMD in Shantou from 2010 to 2015 and obtained 50,361 cases of HFMD. The HFMD in Shantou had significant seasonal characteristics, and the peak of onset was from May to June. This is consistent with some previous studies [27–29]. But the trend of bimodal distribution in Shantou was not significant. In different seasons, climate conditions change, and the optimal climate conditions promote the occurrence and spread of HFMD. HFMD in Shantou mostly occurred among children under 5-years-old.

Among these children, the incidence rate was affected by age, the older the children were, the lower the incidence there would be. Newly-born to 1-year-old children are the main infected population of disease, which was consistent with the results of HFMD research in other areas in China [13, 20, 30]. According to some research, this situation was probably related to the inadequacy of antibodies in the body of children. For example, a recent study found that more than 50% of children under 5-years-old have no neutralising antibody for EV71 and coxA16 [23]. Compared with the scattered children, the incidence of kindergarten children and primary school children was much rarer. The reason for this is that, since 2008, the Chinese Ministry of Health and the local government had taken many measures to control the prevalence of HFMD in the environment of educational institutions, such as disinfection of toys, daily necessities and dining cars, regular inspection, sanitary hand washing intervention, and case isolation [23]. Compared with the educated children, the activities range of the scattered children was bigger, and their health condition was poorer. It was easy to touch the virus and parasitic body with HFMD during activities. Therefore, adverse health conditions might be important reasons for the high incidence of HFMD among scattered children. We also found that the number of boys with HFMD in Shantou was significantly higher than that of girls, and the ratio of male to female in terms of case number was 1.78:1, suggesting that some factors increased the susceptibility or behavioural state of male genes. It was also possible that boys were more active than girls, greatly increasing the chance of exposure to the virus and the spread of HFMD. But this might also be the reporting bias [13].

The incidence rate of HFMD varied widely across the streets of Shantou. Based on our spatial autocorrelation analysis of HFMD in Shantou, except for 2013, there was no spatial clustering in the incidence of HFMD in Shantou, indicating that high incidence areas were more likely to have spatial autocorrelation. There was local spatial autocorrelation in HFMD in Shantou, and the most explicit part was the H-H cluster. In the local area with higher incidence, it was easier to detect the spatial autocorrelation. In this study, a global autocorrelation was only detected in 2013. But in local autocorrelation analysis, the H-H gathering areas were mainly in Queshi street, Zhuchi street and Xinjin street. The incidence in the streets which were around the three high incidence streets was also high. From the geographical location, the high-risk areas of HFMD in Shantou were mainly located in four places: the center of convergence is located in the intersection of Jinping district, Longhu district and Haojiang district, covering the Zhuchi street and Xinjin street and their surroundings. The second was located in the northern Chenghai district and the third in the southern Chaonan district, covering the Hepu street and it surroundings. The forth was in Nan’ao county. The incidence of these regions does not change obviously with time. The above areas of high incidence of HFMD should be the key focus for government departments aiming to prevent and control this disease.

This study has still some limitations. First, spatial autocorrelation analysis of HFMD might explain whether HFMD has a global spatial autocorrelation or local spatial autocorrelation, but in the local spatial autocorrelation, we cannot achieve a clear correlation coefficient. Spatial autocorrelation can only show that the local space has or does not have autocorrelation and whether it has statistical significance, but it doesn’t explain the degree of spatial correlation between streets [6]. At present, the research on the clustering of HFMD mainly takes streets/townships as the smallest descriptive analysis units. If we can get accurate latitude and longitude information of residential quarters and schools in future HFMD space-time scanning analysis research, it will make the analysis of aggregated regional spatial information more detailed, and the prevention and control measures are hopefully more targeted. In addition, due to the different level of doctors in each hospital, the diagnosis of HFMD will be affected, so the data of HFMD may not be completely true. This will seriously affect the reliability of the records, variations in diagnostic sensitivity and specificity of clinician trends in years, and the demographic status of the patients.

## Conclusions

Understanding the incidence and aggregation trend of HFMD can help the government to formulate better prevention and treatment strategies. This study addresses the recent epidemiological characteristics and spatial autocorrelation analysis of HFMD in Shantou. In view of the prevalence of HFMD in Shantou, it is suggested that the Shantou government take measures to protect children under 5 years old from HFMD in spring and summer, especially in May and June. In Shantou, the morbidity varies across streets, and the level of morbidity polarisation is concerning. Therefore, the government should invest more manpower and material resources to prevent and control high-incidence streets than low-incidence streets. At the street scale, HFMD presents a local spatial autocorrelation trend in the Shantou area. According to the results of local autocorrelation analysis, the government should also focus on prevention and control of high incidence areas and vicinities, especially in Queshi street, Zhuchi street and Xinjin street. The prevention and control of these areas with high incidence and high aggregation can effectively prevent the incidence and spreading of HFMD in Shantou.

## Data Availability

The data that support the findings of this study are available Shantou CDC but restrictions apply to the availability of these data, which were used under license for the current study, and so are not publicly available. Data are however available from the authors upon reasonable request and with permission of Shantou CDC. If anyone want to cooperate with Shantou CDC and get data, please contact Shantou CDC staff GXX(E-mail: xuguangxing2005122@163.com).

## Abbreviations

CDC: Center for Disease Control and Prevention
Cox-A16: Coxsackievirus A16
EV71: Enterovirus 71
HFMD: Hand, foot and mouth disease
LISA: Local indicators of spatial association
